# Extent of alignment between the Australian Dietary Guidelines and the NOVA classification system across the Australian packaged food supply

**DOI:** 10.1111/1747-0080.12880

**Published:** 2024-05-13

**Authors:** Hillary Nguyen, Alexandra Jones, Eden M. Barrett, Maria Shahid, Allison Gaines, Monica Hu, Simone Pettigrew, Jason H. Y. Wu, Daisy H. Coyle

**Affiliations:** ^1^ The George Institute for Global Health, University of New South Wales Sydney New South Wales Australia; ^2^ School of Public Health University of California Berkeley California USA; ^3^ Department of Epidemiology and Biostatistics, School of Public Health Imperial College London London UK

**Keywords:** dietary guidelines, food classification, food policy, level of processing, NOVA

## Abstract

**Aims:**

The Australian Dietary Guidelines are currently being revised and ultra‐processed foods have been identified as a high priority action area. To better understand how well the current Dietary Guidelines align with level of processing classifications, the aim of this study was to assess the alignment between the Australian Dietary Guidelines and the NOVA classification system for classifying the healthiness of packaged foods.

**Methods:**

Data were sourced from the Australian FoodSwitch dataset, which included 28 071 packaged food and beverage products available in major Australian supermarkets in 2022. Products were classified as (i) core or discretionary (Australian Dietary Guidelines) and (ii) non‐ultra‐processed or ultra‐processed (NOVA). Agreement between the two systems (core vs. non‐ultra‐processed and discretionary vs. ultra‐processed) was evaluated using the kappa statistic.

**Results:**

There was ‘moderate’ agreement (*κ* = 0.41, 95% CI: 0.40–0.42) between the Australian Dietary Guidelines and the NOVA system, with 69.8% of products aligned across the two systems. Alignment was more common for discretionary foods (80.6% were ultra‐processed) than core foods (59.9% aligned were not‐ultra‐processed). Food categories exhibiting the strongest levels of alignment included confectionary, foods for specific dietary use, and egg and egg products. Discordance was most common for convenience foods, sugars, honey and related products, and cereal and grain products.

**Conclusions:**

Despite moderate alignment between the Australian Dietary Guidelines and NOVA, the discordance observed for almost one‐third of products highlights the opportunity to develop recommendations for ultra‐processed foods within the guidelines to advise Australians how these foods should be considered as part of a healthy diet.

## 1 BACKGROUND

An unhealthy diet, high in sodium and low in whole grains, fruit, nuts and seeds, vegetables and omega‐3 fatty acids, plays a significant role in the development of non‐communicable diseases.^1^ In Australia, dietary risk factors account for approximately 20% of deaths related to non‐communicable diseases, which include those caused by type 2 diabetes, cardiovascular disease and certain types of cancers.^2^ More than 100 countries globally have established dietary guidelines to provide population‐level recommendations on foods, food groups and dietary patterns that are associated with promoting overall health and reducing risk of non‐communicable diseases and obesity.^3^ Dietary guidelines are used by governments to inform national nutrition policies and serve as a resource for health professionals, food manufacturers, educators and researchers to support consumers eat healthier diets.^4^, ^5^

The Australian Dietary Guidelines were last revised in 2013.^6^ These recommend consuming a nutritionally balanced diet consisting of foods from five food groups: (i) vegetables and legumes/beans; (ii) fruit; (iii) grain (cereal) foods, mostly wholegrain and/or high cereal fibre varieties; (iv) lean meats and poultry, fish, eggs, tofu, nuts and seeds, and (v) legumes/beans; milk, yoghurt, cheese and/or alternatives (mostly reduced fat).^6^ These food‐based dietary guidelines also advise people to limit their intake of discretionary foods, which are defined as foods high in saturated fat, added salt and/or added sugars and low in nutritional value.^6^ Research has shown that adherence to the Australian Dietary Guidelines is associated with reduced risk of type 2 diabetes, hypertension and obesity^7^ and improved mental health^8^ and quality of life in older adults (>55 years of age).^9^ The next revision of the Australia Dietary Guidelines is now underway. The current phase of this revision involves reviewing the latest nutrition science research to understand whether the evidence‐base has changed enough since the last round of the guidelines to warrant a change to the dietary recommendations.^10^ Level of processing, specifically ultra‐processing, has been identified as a high priority area of this evidence review.^10^

The growing interest globally in categorising foods based on their level of processing is driven by the mounting evidence linking ultra‐processed foods to a range of adverse health outcomes including weight gain, type 2 diabetes, cardiovascular disease, depression and some types of cancer and all‐cause mortality.^11^, ^12^, ^13^ Ultra‐processed foods and beverages, as defined by the NOVA classification system, are industrially formulated products that are created through various chemical and physical processing techniques.^14^ These products often contain high amounts of refined starches, added salt and added sugars, as well as ingredients unlikely to be used in home kitchens such as cosmetic additives that transform the textural and sensory properties of foods.^14^ The health risks associated with ultra‐processed foods have been mainly attributed to their poor nutritional content^15^, ^16^, ^17^ and their influence on overall diet quality.^16^, ^18^, ^19^, ^20^ However, the evidence also suggests that ultra‐processed foods impact health outcomes through influencing appetite hormones,^21^, ^22^ glycaemic response,^23^ gastric emptying,^24^ and processing and eating rate.^24^

Ultra‐processed foods currently comprise 48% of Australian household grocery purchases (g/d per capita)^25^ and account for 42% of the total energy intake in the average Australian diet.^18^ There is therefore a need to better inform Australians around the health risks associated with these foods. Incorporating level‐of‐processing into the dietary guidelines would bring Australia in line with a growing number of other countries, including Brazil, Belgium, Ecuador and Uruguay, where national guidelines recommend limiting the consumption of ultra‐processed foods.^26^

Considering both the Australian Dietary Guidelines and the NOVA classification system identify the type of products that are healthy (core, non‐ultra‐processed) or unhealthy (discretionary, ultra‐processed), it is possible to assess how these two systems classify foods as healthy or unhealthy according to their different criteria. As such, the aim of this study was to assess the extent of alignment and discordance between the Australian Dietary Guidelines and NOVA for classifying the healthiness of foods across a large representative sample of packaged food and beverages available for sale in Australia. Alignment between the two systems was defined as the Australian Dietary Guidelines classification of core foods aligning with the NOVA classification of non‐ultra‐processed foods and the Australian Dietary Guidelines classification of discretionary foods aligning with the NOVA classification of ultra‐processed foods. By highlighting areas of alignment and divergence between the two systems, the findings will highlight opportunities to enhance cohesion between the national dietary guidelines in Australia and the NOVA classification. These insights are not only valuable for refining dietary recommendations in Australia, but they hold broader significance for other countries worldwide that use food‐based dietary guidelines and are looking to consider level‐of‐processing within their dietary recommendations.

## 2 METHODS

To assess alignment and discordance between the two classification systems, we used data from the 2022 Australian FoodSwitch dataset, a dataset owned and managed by The George Institute for Global Health. FoodSwitch contains brand‐ and product‐specific nutrition information for over 35 000 packaged food and beverage products that were available for sale in 2022 from five large supermarket retailers in Australia. The data is gathered each year by trained collectors who photograph all packaged food products available for sale in‐store. Information about the nutrition composition (per 100 g and per serve), ingredient information, front‐of‐pack labelling and claims information is extracted from the photos and entered into the FoodSwitch dataset.^27^

Products in the FoodSwitch dataset are categorised into a hierarchical system developed by the Global Food Monitoring Group.^28^ This system categorises foods across major categories (e.g., bread and bakery products), categories (e.g., bread) and subcategories (e.g., pita bread). We excluded formulated supplementary foods (meal replacements) and baby and infant foods for children 2 years of age or younger as these are not covered by the Australian Dietary Guidelines.^6^ We also excluded alcohol and vitamins and supplements as they are not covered by the NOVA classification system.

The Australian Dietary Guidelines recommend that Australians consume a wide variety of nutritious foods from the following five food groups each day: (i) vegetables and legumes/beans; (ii) fruit; (iii) grain (cereal) foods, mostly wholegrain and/or high cereal fibre varieties; (iv) lean meats and poultry, fish, eggs, tofu, nuts and seeds; and (v) dairy (milk, yoghurt, cheese and/or their alternatives, mostly reduced fat).^6^ For the purposes of this paper, we refer to foods and beverages that fall within any of these five groups as ‘core’. The Australian Dietary Guidelines also advise that Australians limit their intake of discretionary foods, which include cakes, biscuits, pastries, fried foods, processed meat, chocolate and confectionery.^6^

We classified all products in FoodSwitch as either core or discretionary following a procedure developed by the Australian Bureau of Statistics.^29^ This method was originally designed for the analysis of the 2011–2012 Australian Health Survey and consisted of two key steps. The first step was to assign relevant products as core or discretionary according to the ‘Principles for Identifying Discretionary Foods’ outlined by the Australian Bureau of Statistics.^29^ These principles either apply to an entire food category (e.g., ‘all fruit juices are core, but all other juice drinks are discretionary’) or help to differentiate between products within the same food category (e.g., ‘discretionary foods are defined to be those breakfast cereals with >30 g sugar per 100 g’).^29^ The second step involved matching the remaining foods in FoodSwitch to the comprehensive ‘Discretionary Food List’ also released by the Australian Bureau of Statistics.^30^ This list contains 5740 foods and beverages consumed during the 2011–2012 National Nutrition and Physical Activity Survey and indicates which of these products are discretionary. Any FoodSwitch products that were not defined as ‘discretionary’ were assigned as ‘core’.^30^

The level of processing of foods was determined according to the NOVA classification system, which categorises foods into one of four groups based on the extent, nature and purpose of the industrial processes they undergo, regardless of their final nutrient content: (1) unprocessed or minimally processed foods, (2) processed culinary ingredients, (3) processed foods and (4) ultra‐processed foods.^31^ Products were classified as ultra‐processed if they contained industrial ingredients that are never or rarely used in household kitchens or if they contained additives that are intended to make foods more palatable and/or appealing.^18^, ^25^, ^32^, ^33^ The list of ultra‐processed food ingredients encompassed agents (anti‐caking, firming, glazing, leavening or raising agent); colours; flavours; emulsifiers; extracts; thickeners; any sweeteners other than sugar, maple syrup and honey (e.g., dextrose, fructose, maltitol); components that are inherent in whole foods but included as isolates (e.g., lactose, wheat gluten, triglycerides) and protein powders. Vitamins, minerals and live cultures were not considered cosmetic additives as they are typically used for fortification purposes. Similarly, acids and acidity regulators were not considered cosmetic additives as they are often added as a preservative rather than to alter the flavour or texture.^31^ This methodology has been used in previous research with large datasets^32^ and aligns with recommended approaches suggested by researchers who developed the NOVA classification system.^14^

To apply these methods, we examined the ingredient list of each product in the FoodSwitch database and classified each product as ultra‐processed if it contained at least one cosmetic additive. Although the NOVA system categories non‐ultra‐processed products into three distinct groups (unprocessed/minimally processed, culinary ingredients and processed), we combined them together to form one group for this study to allow for a binary assessment between non‐ultra‐processed (NOVA groups 1–3) and ultra‐processed foods (NOVA group 4).

We conducted cross‐tabulations to compare the classifications of products based on the Australian Dietary Guidelines (core vs. discretionary) with those derived from the NOVA classification system (groups 1–3 vs. group 4). To assess the agreement between the two systems in classifying products as healthier (core or NOVA groups 1–3) or less healthy (discretionary or NOVA group 4), we used the kappa (*κ*) statistic with 95% confidence intervals (CIs). The kappa statistic was selected over a simple percent agreement calculation because it accounts for the possibility of the agreement occurring by chance. The kappa statistic ranges indicate the level of agreement: ≤0 indicates no agreement, 0.01–0.20 indicates ‘slight’ agreement, 0.21–0.40 indicates ‘fair’ agreement, 0.41–0.60 indicates ‘moderate’ agreement, 0.61–0.80 indicates ‘substantial’ agreement and 0.81–0.99 indicates ‘near perfect’ agreement.^34^ We calculated the agreement across all products in the FoodSwitch dataset and also within major categories. For products that were identified as aligned (core and NOVA groups 1–3 or discretionary and NOVA group 4), we calculated the number and proportion (*n*, %) of these products, both overall and within each major category. We also identified the typical contributors to alignment within each major food category. This analysis was then repeated for products that were identified as discordant (core and NOVA group 4 or discretionary and NOVA groups 1–3). All statistical analyses were performed using R.

## 3 RESULTS

Overall, 35 645 food and beverage products were identified in the 2022 FoodSwitch database. After excluding alcohol (*n* = 6556), vitamins and supplements (*n* = 578), baby and infant foods (*n* = 395) and formulated supplementary foods (*n* = 45), 28 071 products were available for the final analysis. According to the Australian Dietary Guidelines, 52.1% (*n* = 14 615) of these products were classified as core and 47.9% (*n* = 13 456) as discretionary. Per the NOVA system, 60.2% (*n* = 16 912) were classified as NOVA group 4 (ultra‐processed) and 39.8% (*n* = 11 159) were classified as NOVA groups 1–3. The number and percent of products across each classification system by major food category are displayed in Tables S1 and S2.

**FIGURE 1:**
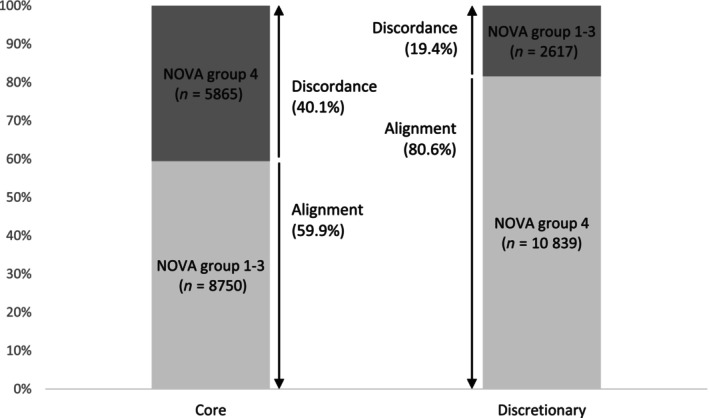
Distribution of products according to the NOVA classification system (groups 1–3 and group 4), separated by the Australian Dietary Guidelines classification of core food products (*n* = 14 615) and discretionary food products (*n* = 13 456). Alignment is defined as core and NOVA groups 1–3 food products and discretionary and NOVA group 4 food products (illustrated in light grey shading). Discordance is defined as core and NOVA group 4 and discretionary and NOVA groups 1–3 food (illustrated in dark grey shading).

**FIGURE 2:**
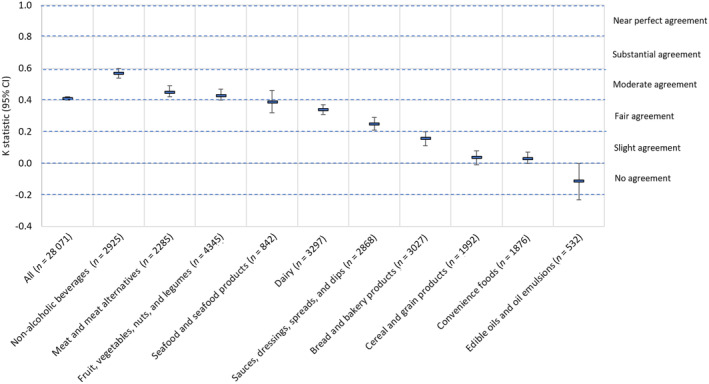
Agreement (*κ*, 95% CI) between the Australian Dietary Guidelines and the NOVA classification system in the proportion of aligned products (core/ NOVA groups 1–3 or discretionary/NOVA group 4) for all products and each major FoodSwitch category. Using the *κ* statistic, ≤0 indicates no agreement (i.e., two systems agree only at chance level); 0.01–0.20 indicates ‘slight’ agreement; 0.21–0.40 indicates ‘fair’ agreement; 0.41–0.60 indicates ‘moderate’ agreement; 0.61–0.80 indicates ‘substantial’ agreement; and 0.81–0.99 indicates ‘near perfect’ agreement. A kappa statistic could not be calculated for five major categories in which all products had the same Australian Dietary Guidelines classification: egg and egg products (all core), confectionery (all discretionary), snack foods (all discretionary), foods for specific dietary use (all discretionary) and sugar, honey and related products categories (all discretionary).

Overall, 69.8% (*n* = 19 589) of all packaged food and beverage products were aligned (Figure 1). This was classified as ‘moderate’ agreement (*κ* = 0.41, 95% CI: 0.40–0.42) between the Australian Dietary Guidelines and NOVA classification system (Figure 2). Discretionary foods were more likely to be aligned with NOVA group 4 (80.6% of discretionary foods, *n* = 10 839) and core foods were more likely to be aligned with NOVA groups 1–3 (59.9% of core foods, *n* = 8750). However, one‐third of the sample (30.2%, *n* = 8482) consisted of discordant products; 40.1% (*n* = 5865) of core foods were classified as NOVA group 4 and 19.4% (*n* = 2617) of discretionary foods were classified as NOVA groups 1–3 (Figure 1).

Within the major food categories, ‘moderate’ agreement (*κ* = 0.41) was found between the Australian Dietary Guidelines and the NOVA classification system for three categories: non‐alcoholic beverages (*κ* = 0.57), meat and meat alternatives (*κ* = 0.45) and fruit, vegetables, nuts and legumes (*κ* = 0.43). ‘Fair’ agreement was found for three categories including seafood and seafood products (*κ* = 0.39), dairy (*κ* = 0.34) and sauces, dressings, spreads and dips (*κ* = 0.25). ‘Slight’ agreement was found for three categories including bread and bakery products (*κ* = 0.16), cereal and grain products (*κ* = 0.04) and convenience foods (*κ* = 0.03). There was no agreement for edible oils and oil emulsions (*κ* = −0.11) (Figure 2). A kappa statistic could not be calculated for five major categories in which all products had the same Australian Dietary Guidelines classification: egg and egg products (all core), confectionery (all discretionary), snack foods (all discretionary), foods for specific dietary use (all discretionary) and sugar, honey and related products categories (all discretionary). While these five categories all belonged to the same Australian Dietary Guidelines category, there was variation in their alignment with NOVA as discussed below.

The overall alignment between the two systems varied across major food categories, ranging from 35.1% to 96.1%. Confectionary demonstrated the highest alignment (96.1%) followed by foods for specific dietary use (95.7%) and egg and egg products (95.6%) (Table S3). Within the core and NOVA groups 1–3 products, the relative proportion of aligned products ranged from 0.0% to 95.6%. Food categories with the highest relative proportion of aligned products were eggs and egg products (95.6%), fruits, vegetables and other legumes (66.2%) and seafood and seafood products (59.7%) (Table 1). For discretionary and NOVA group 4 products, the proportion of aligned products ranged from 0.0% to 96.1% across the major food categories. Confectionery foods exhibited the highest alignment (96.1%), followed by foods for specific dietary use (95.4%) and snack foods (82.1%) (Table 1).

**TABLE 1:** Number (*n*) and percent (%) of aligned products (core food products classified as NOVA groups 1–3 and discretionary foods classified as NOVA group 4) within each major food category and typical contributors to alignment.

Major food category	*N*	Core and NOVA groups 1–3	Typical contributors to alignment	Discretionary and NOVA group 4	Typical contributors to alignment
Bread and bakery products	3027	235 (7.8)	Freshly baked bread, sourdough bread, plain crispbread, pita bread/wraps, pizza bases, plain rice crackers	1808 (59.7)	Biscuits (cookies), cake and cake mixes, baked goods (e.g., croissant, doughnuts, muffins), garlic bread, flavoured crackers and crispbread
Cereal and grain products	1992	986 (49.5)	Flour (all types), plain pasta, plain noodles, raw plain rice (all types), plain oats, muesli/granola	41 (2.1)	Sweet and extruded cereals (e.g., cocoa‐based), stuffing mixes
Confectionery	1798	0 (0.0)	None	1727 (96.1)	Some chocolate (plain, or with nuts/fruit), sweet confectionary (e.g., jelly beans, snacks), chewing gum, fudge, fairy floss, liquorice, marshmallows, mints
Convenience foods	1876	296 (15.8)	Chilled and canned soups and fresh, chilled and frozen ready meals, ready‐made salads	363 (19.3)	Chilled and frozen ready meals, frozen pastries, pizza, dry soup mixes
Dairy	3297	1358 (41.2)	Cheddar cheese and other similar cheeses, camembert/brie, plain dairy milk, plain dairy yoghurt	769 (23.3)	Ice‐cream, condensed milk, frozen yoghurt, rice pudding, mousse, thickened cream, dairy desserts (e.g., pannacotta)
Edible oils and oil emulsions	532	291 (54.7)	Nut and vegetable oils (e.g., olive, rice brain, sesame, coconut, avocado)	21 (3.9)	Salted and unsalted butter spreads, flavoured butter spreads (e.g., garlic)
Egg and egg products	90	86 (95.6)	Fresh eggs, plain egg whites	0 (0.0)	None
Foods for specific dietary use	369	0 (0.0)	None	353 (95.4)	Protein bars, protein powders, protein drinks, protein balls
Fruit, vegetables, nuts and legumes	4345	2877 (66.2)	Packaged fruits and vegetables (fresh, frozen), canned fruit, canned vegetables and legumes, herbs and spices, dried legumes, nuts (salted and unsalted), dried fruit	466 (10.7)	Seasonings, jams, frozen chips/wedges, hash browns, jam/marmalades, fruit bars/bites
Meat and meat alternatives	2285	695 (30.4)	Raw unflavoured meat (e.g., beef, chicken, lamb, pork), plant‐based meat products, falafel, tofu	956 (41.8)	Meat pies, sausage rolls, sausages, canned meat, crumbed chicken products (e.g., nuggets), hot dogs
Non‐alcoholic beverages	2925	1154 (39.5)	Instant coffee and coffee beans, plain tea (e.g., black and herbal), plain water, fruit and vegetable juices	1120 (38.3)	Soft drinks (both diet and sugar‐sweetened), flavoured coffee, juice drinks, flavoured water, cordials, energy drinks
Sauces, dressings, spreads and dips	2868	269 (9.4)	Tomato paste, herb pastes, peanut and other nut butters	1771 (61.8)	Condiments (e.g., BBQ sauce, mayonnaise, chutney), salad dressings, pasta sauces, meal‐based sauces, chocolate spreads, dips, marinades, gravy, stock
Seafood and seafood products	842	503 (59.7)	Canned fish, chilled and frozen seafood	122 (14.4)	Crumbed and battered seafood
Snack foods	1284	0 (0.0)	None	1054 (82.1)	Muesli bars, flavoured potato and corn crisps, extruded snacks
Sugars, honey and related products	541	0 (0.0)	None	269 (49.7)	Sprinkles, sweet sauces (e.g., caramel/strawberry sauce), icing/frosting, low calorie sweeteners, cake/cupcake decorations

The overall discordance across major food categories ranged from 3.9% to 64.9%. The highest level of discordance was found for convenience foods (64.9%), followed by sugars, honey and related products (50.3%) and cereal and grain products (48.4%). For core products classified as NOVA group 4, the relative proportion of discordance ranged from 0.0% to 61.8%, with convenience foods (61.8%), cereal and grain products (48.4%) and dairy (33.8%) exhibiting the highest proportions of discordance (Table 2). For discretionary and NOVA groups 1–3 products, the relative proportion of discordant products ranged from 0.0% to 50.3%. The major food categories with the highest relative discordance included sugars, honey and related products (50.3%), sauces, dressings, spreads and dips (25.1%) and edible oils and oil emulsions (21.8%) (Table 2).

**TABLE 2:** Number (*n*) and percent (%) of discordant products (core food products classified as NOVA group 4 and discretionary foods classified as NOVA groups 1–3) within each major food category and typical contributors to discordance.

Major food category	*N*	Core and NOVA group 4	Typical contributors to discordance	Discretionary and NOVA groups 1–3	Typical contributors to discordance
Bread and bakery products	3027	802 (26.5)	Bagels, prepackaged bread, fruit bread, crumpets, prepackaged pizza bases, flavoured rice crackers, wraps and tortillas, pancake/pikelet mixes	182 (6.0)	Grain‐based crackers and crispbread, shortbread biscuits (cookies), breadsticks, filo pastry
Cereal and grain products	1992	965 (48.4)	Flavoured rice, microwavable rice (all types), bread mixes, breadcrumbs, canned pasta, ravioli, instant noodles, flavoured oats, breakfast flakes/bran/biscuits	0 (0.0)	None
Confectionery	1798	0 (0.0)	None	71 (3.9)	Some chocolate (plain, or with nuts/fruit), crystallised ginger, cacao nibs
Convenience foods	1876	1159 (61.8)	Chilled and canned soups and fresh, chilled and frozen ready meals, ready‐made salads, frozen dumplings, sushi, sandwiches and rolls, pizza	58 (3.1)	Fresh and chilled ready meals and some vegetarian snack foods
Dairy	3297	1114 (33.8)	Flavoured milk, flavoured yoghurt, dairy free cheese, processed cheese, plant‐based milk	56 (1.7)	Sour cream, fresh and thickened cream, condensed milk
Edible oils and oil emulsions	532	104 (19.5)	Margarines, cooking oils sprays	116 (21.8)	Salted and unsalted butter, ghee, animal fat
Egg and egg products	90	4 (4.4)	Scrambled egg mixes, vegan egg replacers	0 (0.0)	None
Foods for specific dietary use	369	0 (0.0)	None	16 (4.3)	Nut and dried fruit based protein bars and balls
Fruit, vegetables, nuts and legumes	4345	317 (7.3)	Canned baked beans, salad kit, fruit purees	685 (15.8)	Frozen potato chips and mash, 100% fruit straps and fruit bites, olives, pickled vegetables, seasoning mixes, plain salt
Meat and meat alternatives	2285	508 (22.2)	Plant‐based meat products, seasoned meats (e.g., chicken, pork, beef, lamb)	126 (5.5)	Bacon, salami, pancetta, prosciutto, dried meats (e.g., jerky)
Non‐alcoholic beverages	2925	570 (19.5)	Fruit and vegetable juices, breakfast beverages	81 (2.8)	Kombucha, juice drinks
Sauces, dressings, spreads and dips	2868	109 (3.8)	Peanut butters, herb paste, tomato paste	719 (25.1)	Pasta sauces, dips, curry pastes, vinegars, pesto, salsas, salad dressings, relish
Seafood and seafood products	842	213 (25.3)	Canned fish, crumbed and battered seafood, fish spread	5 (0.6)	Crumbed seafood
Snack foods	1284	0 (0.0)	None	230 (17.9)	Plain potato and corn crisps, legume‐based snacks, seaweed snacks
Sugars, honey and related products	541	0 (0.0)	None	272 (50.3)	Honey, sugar (brown, white), syrups (golden, maple, rice malt), gelatine, natural food/herb essences

## 4 DISCUSSION

This study used a large and nationally representative sample of the Australian packaged food supply to quantify the extent of alignment between the Australian Dietary Guidelines and the NOVA food system for classifying the healthiness of packaged foods. Overall, the results showed moderate agreement, with almost 70% of products aligned between the two systems. Alignment was more common for the classification of discretionary and ultra‐processed foods than for core and non‐ultra‐processed foods, suggesting there is less consistency between the two systems for what constitutes a healthy recommended food than for what constitutes an unhealthy food. Overall, alignment was strongest for confectionary foods, foods for specific dietary use and egg and egg products while discordance was most common for convenience foods, sugars, honey and related products and cereal and grain products. These findings highlight that if the upcoming revision to the Australian Dietary Guidelines aims to integrate the concept of level of processing into its dietary recommendations, changes would be required with respect to what foods are recommended as healthy and what are classified as discretionary.

In this study, there was moderate overall alignment between the Australian Dietary Guidelines and the NOVA food classification system. While one in three products were found to be discordant across the two systems, this misalignment was not heavily concentrated to specific food categories but rather distributed across all categories. This suggests that incorporation of NOVA in the Australian Dietary Guidelines may be best achieved through nuanced changes to the guidelines that can help to differentiate between products within different food categories according to level of processing. The main changes required to better align the Australian Dietary Guidelines and NOVA are described in detail below.

The most common type of discordance was found for core foods that are ultra‐processed. Included within this group were flavoured milks, flavoured yoghurt, margarine and pikelets. It is questionable whether these foods should be recommended within the Australian Dietary Guidelines as part of a healthy diet, especially considering many are not only ultra‐processed but high in added sugars and/or saturated fat, which is in direct conflict with a recommendation in the current dietary guidelines, which states to ‘limit intake of foods containing saturated fat, added salt, added sugars and alcohol’.^6^ If level of processing were to be incorporated within the next edition of the Australian Dietary Guidelines, this could mean that re‐classification of a number of foods groups, including flavoured milk and yoghurt, from a recommended food to a discretionary food, would need to be considered.

The second type of discordant products (core but ultra‐processed) are foods that happen to be ultra‐processed due to the presence of at least one ingredient that is considered a marker of ultra‐processing but belongs to a larger food category that also contains non‐ultra‐processed alternatives. These foods include pre‐packaged wholegrain breads and some ready‐to‐eat foods including salads and ready meals. The fact that non‐ultra‐processed versions of these foods exist, for example, fresh wholegrain bread and ready‐to‐eat foods free from ultra‐processed food ingredients, suggests versions of these foods can be produced and formulated in way that does not require ultra‐processing. Versions of these foods could be promoted in the guidelines. However, the distinction between ultra‐processed and non‐ultra‐processed versions of these foods based on the presence of certain additives also reflects a criticism of NOVA in the literature in that it categorises products too broadly and does not adequately account for the nutritional composition of foods.^35^ This means that not all ultra‐processed foods are necessarily unhealthy according to their nutrient profile. Moreover, many food processing techniques are used to promote nutrient availability and preserve foods for long‐term consumption or for increasing food access in remote areas or certain populations.^36^ Dealing with the complexity of differentiating between ultra‐processed and non‐ultra‐processed core foods within a specific food category while simultaneously considering the nature of the dietary guidelines, which are designed to provide guidance at a food category level, will need to be dealt with if the next revision of the Australian Dietary Guidelines incorporates level of processing. One potential solution could be to advise consumers to opt for non‐ultra‐processed versions of foods where available or choose home‐cooked or fresh products over processed and prepackaged options.^6^ This approach would align with dietary guidelines implemented in 18 countries globally that have now integrated processing‐related terms within their guidelines, including terminology around limiting ‘ultra‐processed foods’, ‘highly‐processed foods’ and ‘processed foods’.^37^

In the case of discretionary and non‐ultra‐processed foods, we found similar drivers for discordance as raised above. Many discretionary food categories contained both ultra‐processed and non‐ultra‐processed foods due to the presence or absence of ultra‐processed ingredients. For example, some dips, pesto, plain potato chips, corn chips and shortbread biscuits contained no ingredients that are markers of ultra‐processing despite many of these products falling within a food category that largely contains ultra‐processed foods. These food categories are considered discretionary due to being nutrient‐poor and high in salt, added sugars and saturated fat. While these foods may be appropriately captured as discretionary due to their poor nutritional profile, it is important to note that not all versions of these foods are ultra‐processed. If level of processing is incorporated into the dietary guidelines, this could create some confusion for consumers about whether non‐ultra‐processed versions of these foods are a healthier choice. Further investigation into the health impact of different food ingredients and processing methods within these product categories is required to properly understand whether the omission of additives and cosmetic ingredients in these products offsets some of the negative effects of their nutritional profile. Such research would be useful in understanding whether discretionary foods should account for level of processing beyond the nutritional composition alone.

The final group of discordant products are some single‐ingredient discretionary foods also known as processed culinary ingredients according to NOVA (NOVA group 2). Typical products included within the category included honey, sugars, natural syrups, butter and animal fats. These foods are categorised as processed culinary ingredients due to the fact they are often produced from minimally processed foods (NOVA group 1) but processed to some degree and used in preparation, seasoning and cooking of minimally processed foods rather than consumed alone.^38^ This definition reflects how these foods are used and how they are consumed in their final form rather than how healthy they are in isolation, and as such does not align with the food‐based classification system used in the Australian Dietary Guidelines.^6^ While the poor nutritional quality of these products makes them suitable for discretionary food status, their classification as a discretionary food despite not being ultra‐processed may need to be acknowledged and addressed if level of processing is considered within the next round of the Australian Dietary Guidelines.

In line with prior research,^39^, ^40^ the present research findings highlight the domination of unhealthy and ultra‐processed packaged foods available for sale in Australian supermarkets. The focus on ultra‐processed foods as a key priority of the evidence review for the next revision of the Australian Dietary Guidelines^10^ is an encouraging step towards nutrition policies in Australia acknowledging the health impact of ultra‐processed foods.^11^, ^12^, ^13^ Provision of guidance around ultra‐processed food consumption within the dietary guidelines may also prompt further policy actions targeting these foods. Examples of such actions could include front‐of‐pack labels that identify ultra‐processed foods, restrictions on advertising and/or taxes for ultra‐processed foods.^41^ There is a precedent for this—the current national dietary guidelines,^6^ which recommended Australians ‘limit intake of foods containing added sugars’, prompted Food Ministers to call for changes to added sugar claims^42^ and added sugar labelling^43^ to better guide Australians towards reducing their consumption of foods high in added sugars. As part of the next revision of the Australian Dietary Guidelines, it is also important that consideration is given to nutrient composition when classifying the healthiness of foods to ensure the recommendations for healthy and unhealthy foods align with other existing nutrition schemes such as the Health Star Rating.^32^

The strengths of this study include the large number of included packaged food products that represent the contemporary Australian food supply. Our systematic process of identifying NOVA group 4 products at the individual product level rather than subcategory level is not only the recommended approach to applying NOVA but it also allowed for insights into the characteristics of specific discordant products. Moreover, this study investigated contributors to both aligned and misaligned products when comparing the Australian Dietary Guidelines against the NOVA classification system. Not only does this investigation align with a priority area of National Health and Medical Research Council for the next revision of the Australian Dietary Guidelines^10^ but it also provides a detailed understanding of how the existing dietary guidelines would need to be revised to reflect level of processing within its recommendations.

Some limitations need to be acknowledged. The core and discretionary food classification used for this project was initially generated for ~5700 food products.^30^ We adapted this classification for our work in order to apply it to over 28 000 unique foods. As a result, it is possible that some packaged foods might have been misclassified. However, to reduce this risk, two researchers classified products independently and a third researcher checked any discrepancies. As the FoodSwitch dataset contains only information on the packaged food supply, we were unable to assess compliance between the two systems for unpackaged products such as fresh fruits and vegetables, fresh bakery products and deli items. It is also important to acknowledge this paper compares two classifications that are founded on different principles. The NOVA system is a specific method for classifying foods, whereas the Australian Dietary Guidelines do not represent a classification system per se, but rather are comprehensive guidelines that include both qualitative and quantitative food‐based dietary advice for different populations. In this paper, we have used the Australian Bureau of Statistics tool to classify packaged foods according to the Australian Dietary Guidelines (to allow for comparison to NOVA), and by doing so, we have not captured the scope of the Australian Dietary guidelines in their entirety. Lastly, the results may not be generalisable or relevant to all countries, especially those without national dietary guidelines or those that already incorporate level of processing into their national dietary guideline recommendations.

Our analysis of over 28 000 packaged food and beverage products available on the Australian market revealed that the current Australian Dietary Guidelines and the NOVA classification system are aligned for almost 70% of foods. Despite this moderate alignment, the discordance observed for almost one‐third of products highlights the opportunity for more consistency across the two systems for the foods recommended for a healthy diet and those that should be limited. Providing guidance on ultra‐processed foods within national Dietary Guidelines in Australia would be an important step towards advising Australians how these foods could be consumed as part of a healthy diet.

## AUTHOR CONTRIBUTIONS

HN, AJ, EMB and DHC designed the research; HN and DHC conducted the research; HN and DHC analysed data, HN and DHC wrote the paper; DHC had primary responsibility for final content. All authors provided critical feedback on the manuscript and read and approved the final manuscript.

## FUNDING INFORMATION

This work was supported by an NHMRC Investigator Grant (APP2026320) The content is solely the responsibility of the authors and does not necessarily reflect the official views of the NHMRC.

## CONFLICT OF INTEREST STATEMENT

The authors declare no conflicts of interest.

## Supporting information


**Data S1.** Supporting Information.

## Data Availability

The data that support the findings of this study are available from The George Institute for Global Health. Restrictions apply to the availability of these data, which were used under license for this study. Data are available from the author(s) with the permission of The George Institute for Global Health.

## References

[1] Afshin A , Sur PJ , Fay KA , et al. Health effects of dietary risks in 195 countries, 1990‐2017: a systematic analysis for the Global Burden of Disease Study 2017. Lancet. 2019;393(10184):1958‐1972. 30954305 10.1016/S0140-6736(19)30041-8 PMC6899507

[2] Melaku YA , Renzaho A , Gill TK , et al. Burden and trend of diet‐related non‐communicable diseases in Australia and comparison with 34 OECD countries, 1990‐2015: findings from the Global Burden of Disease Study 2015. Eur J Nutr. 2019;58(3):1299‐1313. 29516222 10.1007/s00394-018-1656-7

[3] Food and Agriculture Organization of the United Nations . Food‐based dietary guidelines: FAO. 2023. Available from: https://www.fao.org/nutrition/education/food‐dietary‐guidelines/regions/en/

[4] Wijesinha‐Bettoni R , Khosravi A , Ramos AI , et al. A snapshot of food‐based dietary guidelines implementation in selected countries. Glob Food Sec. 2021;29:100533.

[5] Wingrove K , Lawrence MA , Russell C , McNaughton SA . Evidence use in the development of the Australian dietary guidelines: a qualitative study. Nutrients. 2021;13(11):1‐17. 10.3390/nu13113748 PMC8620517 34836004

[6] National Health and Medical Research Council . Australian Dietary Guidelines Summary Canberra: National Health and Medical Research Council. 2013. Available from: https://www.eatforhealth.gov.au/sites/default/files/2022‐09/n55a_australian_dietary_guidelines_summary_131014_1.pdf

[7] Livingstone KM , McNaughton SA . Diet quality is associated with obesity and hypertension in Australian adults: a cross sectional study. BMC Public Health. 2016;16(1):1037. 27716133 10.1186/s12889-016-3714-5 PMC5045600

[8] Huddy RL , Torres SJ , Milte CM , McNaughton SA , Teychenne M , Campbell KJ . Higher adherence to the Australian Dietary Guidelines is associated with better mental health status among Australian adult first‐time mothers. J Acad Nutr Diet. 2016;116(9):1406‐1412. 26947337 10.1016/j.jand.2016.01.010

[9] Gopinath B , Russell J , Flood VM , Burlutsky G , Mitchell P . Adherence to dietary guidelines positively affects quality of life and functional status of older adults. J Acad Nutr Diet. 2014;114(2):220‐229. 24239401 10.1016/j.jand.2013.09.001

[10] National Health and Medical Research Council . Priority research questions Canberra: NHMRC. 2023. Available from: https://www.nhmrc.gov.au/health-advice/nutrition/australian-dietary-guidelines-review/priority-research-questions

[11] Pagliai G , Dinu M , Madarena M , Bonaccio M , Iacoviello L , Sofi F . Consumption of ultra‐processed foods and health status: a systematic review and meta‐analysis. Br J Nutr. 2021;125:308‐318. 32792031 10.1017/S0007114520002688 PMC7844609

[12] Juul F , Martinez‐Steele E , Parekh N , Monteiro C , Chang V . Ultra‐processed food consumption and excess weight among US adults. Br J Nutr. 2018;120(1):90‐100. 29729673 10.1017/S0007114518001046

[13] Chen X , Zhang Z , Yang H . Consumption of ultra‐processed foods and health outcomes: a systematic review of epidemiological studies. Nutr J. 2020;19(86):86. 32819372 10.1186/s12937-020-00604-1 PMC7441617

[14] Monteiro CA , Cannon G , Levy RB , et al. Ultra‐processed foods: what they are and how to identify them. Public Health Nutr. 2019;22(5):936‐941. 30744710 10.1017/S1368980018003762 PMC10260459

[15] Poti J , Mendez M , Ng S , Popkin B . Is the degree of food processing and convenience linked with the nutritional quality of foods purchased by US households? Am J Clin Nutr. 2015;101(6):1251‐1262. 25948666 10.3945/ajcn.114.100925 PMC4441809

[16] Martínez Steele E , Baraldi L , Louzada M . Ultra‐processed foods and added sugars in the US diet: evidence from a nationally representative cross‐sectional study. BMJ Open. 2016;6(3):e009892. 10.1136/bmjopen-2015-009892 PMC4785287 26962035

[17] Luiten C , Steenhuis I , Eyles H , Ni Mhurchu C , Waterlander W . Ultra‐processed foods have the worst nutrient profile, yet they are the most available packaged products in a sample of New Zealand supermarkets. Public Health Nutr. 2016;19(3):530‐538. 26222226 10.1017/S1368980015002177 PMC10271194

[18] Machado P , Steele E , Levy R . Ultra‐processed foods and recommended intake levels of nutrients linked to non‐communicable diseases in Australia: evidence from a nationally representative crosssectional study. BMJ Open. 2019;9:e029544. 10.1136/bmjopen-2019-029544 PMC6720475 31462476

[19] Moubarac J‐C , Batal M , Louzada M . Consumption of ultra‐processed foods predicts diet quality in Canada. Appetite. 2017;108:512‐520. 27825941 10.1016/j.appet.2016.11.006

[20] Liu J , Steele E , Karageorgou D , Micha R , Monteiro C , Mozaffarian D . Consumption of ultra‐processed foods and diet quality among U.S. adults and children. Am J Prev Med. 2020;4:4. 10.1016/j.amepre.2021.08.014 PMC9384846 34753645

[21] Hall KD , Ayuketah A , Brychta R , et al. Ultra‐processed diets cause excess calorie intake and weight gain: an inpatient randomized controlled trial of ad libitum food intake. Cell Metab. 2019;30(1):67‐77. 31105044 10.1016/j.cmet.2019.05.008 PMC7946062

[22] Crimarco A , Landry MJ , Gardner CD . Ultra‐processed foods, weight gain, and Co‐morbidity risk. Curr Obes Rep. 2022;11(3):80‐92. 34677812 10.1007/s13679-021-00460-y PMC8532572

[23] Juul F , Vaidean G , Parekh N . Ultra‐processed foods and cardiovascular diseases: potential mechanisms of action. Adv Nutr. 2021;12(5):1673‐1680. 33942057 10.1093/advances/nmab049 PMC8483964

[24] Valicente VM , Peng CH , Pacheco KN , et al. Ultraprocessed foods and obesity risk: a critical review of reported mechanisms. Adv Nutr. 2023;14(4):718‐738. 37080461 10.1016/j.advnut.2023.04.006 PMC10334162

[25] Coyle DH , Huang L , Shahid M , et al. Socio‐economic difference in purchases of ultra‐processed foods in Australia: an analysis of a nationally representative household grocery purchasing panel. Int J Behav Nutr Phys Act. 2022;19(1):148. 36503612 10.1186/s12966-022-01389-8 PMC9742014

[26] Koios D , Machado P , Lacy‐Nichols J . Representations of ultra‐processed foods: a global analysis of how dietary guidelines refer to levels of food processing. Int J Health Policy Manag. 2022;11(11):2588‐2599. 35184508 10.34172/ijhpm.2022.6443 PMC9818109

[27] Dunford E , Trevena H , Goodsell C , et al. FoodSwitch: a Mobile phone app to enable consumers to make healthier food choices and crowdsourcing of national food composition data. JMIR Mhealth Uhealth. 2014;2(3):e37. 25147135 10.2196/mhealth.3230 PMC4147708

[28] Dunford E , Webster J , Metzler AB , et al. International collaborative project to compare and monitor the nutritional composition of processed foods. Eur J Prev Cardiol. 2012;19(6):1326‐1332. 21971487 10.1177/1741826711425777

[29] Australian Bureau of Statistics . Discretionary foods Canberra: ABS. 2015. Available from: https://www.abs.gov.au/ausstats/abs@.nsf/lookup/4363.0.55.001chapter65062011-13

[30] Australian Bureau of Statistics . Australian health survey ‐ discretionary food list. Canberra: ABS. 2018.

[31] Monteiro CA , Cannon G , Lawrence M , Costa Louzada ML , Machado PP . Ultra‐Processed Foods, Diet Quality, and Health Using the NOVA Classification System. FAO; 2019.

[32] Barrett EM , Gaines A , Coyle DH , et al. Comparing product healthiness according to the Health Star Rating and the NOVA classification system and implications for food labelling systems: an analysis of 25 486 products in Australia. Nutr Bull. 2023;48:523‐534. 37897130 10.1111/nbu.12640

[33] Dickie S , Woods J , Machado P , Lawrence M . A novel food processing‐based nutrition classification scheme for guiding policy actions applied to the Australian food supply. Front Nutr. 2023;10:1071356. 36742430 10.3389/fnut.2023.1071356 PMC9895835

[34] Viera AJ , Garrett JM . Understanding interobserver agreement: the kappa statistic. Fam Med. 2005;37(5):360‐363. 15883903

[35] Petrus RR , do Amaral Sobral PJ , Tadini CC , Gonçalves CB . The NOVA classification system: a critical perspective in food science. Trends Food Sci Technol. 2021;116:603‐608.

[36] Weaver CM , Dwyer J , Fulgoni VL 3rd , et al. Processed foods: contributions to nutrition. Am J Clin Nutr. 2014;99(6):1525‐1542. 24760975 10.3945/ajcn.114.089284 PMC6410904

[37] Anastasiou K , Ribeiro De Melo P , Slater S , et al. From harmful nutrients to ultra‐processed foods: exploring shifts in ‘foods to limit’ terminology used in national food‐based dietary guidelines. Public Health Nutr. 2022;26:1‐12. 36458692 10.1017/S1368980022002580 PMC10641640

[38] Monteiro CCC , Lawrence M , Costa Louzada M , Pereira Machado P . Ultra‐processed foods, diet quality, and health using the NOVA classification system. 2019.

[39] Dickie S , Woods JL , Baker P , Elizabeth L , Lawrence MA . Evaluating nutrient‐based indices against food‐ and diet‐based indices to assess the health potential of foods: how does the Australian Health Star Rating system perform after five years? Nutrients. 2020;12(5):1463. 32443570 10.3390/nu12051463 PMC7284529

[40] Menday H , Neal B , Wu JHY , Crino M , Baines S , Petersen KS . Use of added sugars instead of total sugars may improve the capacity of the Health Star Rating system to discriminate between core and discretionary foods. J Acad Nutr Diet. 2017;117(12):1921‐1930.e11. 29173348 10.1016/j.jand.2017.08.013

[41] Popkin BM , Barquera S , Corvalan C , et al. Towards unified and impactful policies to reduce ultra‐processed food consumption and promote healthier eating. Lancet Diabetes Endocrinol. 2021;9(7):462‐470. 33865500 10.1016/S2213-8587(21)00078-4 PMC8217149

[42] Food Standards Australia and New Zealand . P1062 defining added sugars for claims: FSANZ. 2023. Available from: https://www.foodstandards.gov.au/code/proposals/Pages/P1062‐Defining‐added‐sugars‐for‐claims.aspx

[43] Food Standards Australia and New Zealand . Proposal P1058—Nutrition labelling about added sugars: FSANZ. 2023. Available from: https://www.foodstandards.gov.au/code/proposals/Pages/Proposal‐P1058‐‐‐Nutrition‐labelling‐about‐added‐sugars.aspx

