# Supplementary material for: Extent of alignment between the Australian Dietary Guidelines and the NOVA classification system across the Australian packaged food supply
Source: Nutr Diet. 2024 May 13;82(1):42–52. doi: 10.1111/1747-0080.12880 (PMC11795230; doi:10.1111/1747-0080.12880)
Supplement: Supplementary file 1 — Data S1. Supporting Information. [file NDI-82-42-s001.docx]

**Supplementary Table 1.** Number (n) and percentage (%) of core and discretionary food products overall and across each of the major FoodSwitch categories.

| **Major food category** | **Total**  **N** | **Australian Dietary Guidelines** | |
| --- | --- | --- | --- |
|  |  | **Core** | **Discretionary** |
| **All food categories combined** | 28,071 | 14,615 (52.1) | 13,456 (47.9) |
| Bread and bakery products | 3,027 | 1,037 (34.3) | 1,990 (65.7) |
| Cereal and grain products | 1,992 | 1,951 (97.9) | 41 (2.1) |
| Confectionery | 1,798 | 0 (0.0) | 1,798 (100.0) |
| Convenience foods | 1,876 | 1,455 (77.6) | 421 (22.4) |
| Dairy | 3,297 | 2,472 (75.0) | 825 (25.0) |
| Edible oils and oil emulsions | 532 | 395 (74.2) | 137 (25.8) |
| Egg and egg products | 90 | 90 (100.0) | 0 (0.0) |
| Foods for specific dietary use | 369 | 0 (0.0) | 369 (100.0) |
| Fruits, vegetables, nuts and legumes | 4,345 | 3,194 (73.5) | 1,151 (26.5) |
| Meat and meat alternatives | 2,285 | 1,203 (52.6) | 1,082 (47.4) |
| Non-alcoholic beverages | 2,925 | 1,724 (58.9) | 1,201 (41.1) |
| Sauces, dressings, spreads and dips | 2,868 | 378 (13.2) | 2,490 (86.8) |
| Seafood and seafood products | 842 | 716 (85.0) | 126 (15.0) |
| Snack foods | 1,284 | 0 (0.0) | 1,284 (100.0) |
| Sugars, honey and related products | 541 | 0 (0.0) | 541 (100.0) |

**Supplementary Table 2.** Number (n) and percentage (%) of NOVA group 1-3 and NOVA group 4 products overall and across each of the major FoodSwitch categories.

| **Major food category** | **Total**  **N** | **NOVA** | |
| --- | --- | --- | --- |
|  |  | **Group 1-3** | **Group 4** |
| **All food categories combined** | 28,071 | 11,159 (39.8) | 16,912 (60.2) |
| Bread and bakery products | 3,027 | 417 (13.8) | 2,610 (86.2) |
| Cereal and grain products | 1,992 | 986 (49.5) | 1,006 (50.5) |
| Confectionery | 1,798 | 71 (3.9) | 1,727 (96.1) |
| Convenience foods | 1,876 | 354 (18.9) | 1,522 (81.1) |
| Dairy | 3,297 | 1,414 (42.9) | 1,883 (57.1) |
| Edible oils and oil emulsions | 532 | 407 (76.5) | 125 (23.5) |
| Egg and egg products | 90 | 86 (95.6) | 4 (4.4) |
| Foods for specific dietary use | 369 | 16 (4.3) | 353 (95.7) |
| Fruits, vegetables, nuts and legumes | 4,345 | 3,562 (82.0) | 783 (18.0) |
| Meat and meat alternatives | 2,285 | 821 (35.9) | 1,464 (64.1) |
| Non-alcoholic beverages | 2,925 | 1,235 (42.2) | 1,690 (57.8) |
| Sauces, dressings, spreads and dips | 2,868 | 988 (34.4) | 1,880 (65.6) |
| Seafood and seafood products | 842 | 508 (60.3) | 334 (39.7) |
| Snack foods | 1,284 | 230 (17.9) | 1,054 (82.1) |
| Sugars, honey and related products | 541 | 272 (50.3) | 269 (49.7) |

**Supplementary Table 3**. Number (n) and percent (%) of aligned and discordant within each major food category

| **Major food category** | **Total N** | **Aligned**  **products** | | **Discordant products** | |
| --- | --- | --- | --- | --- | --- |
|  |  | **N** | **%** | **N** | **%** |
| Bread and bakery products | 3027 | 2043 | 67.5 | 984 | 32.5 |
| Cereal and grain products | 1992 | 1027 | 51.6 | 965 | 48.4 |
| Confectionery | 1798 | 1727 | 96.1 | 71 | 3.9 |
| Convenience foods | 1876 | 659 | 35.1 | 1217 | 64.9 |
| Dairy | 3297 | 2127 | 64.5 | 1170 | 35.5 |
| Edible oils and oil emulsions | 532 | 312 | 58.6 | 220 | 41.4 |
| Egg and egg products | 90 | 86 | 95.6 | 4 | 4.4 |
| Foods for specific dietary use | 369 | 353 | 95.7 | 16 | 4.3 |
| Fruit, vegetables, nuts and legumes | 4345 | 3343 | 76.9 | 1002 | 23.1 |
| Meat and meat alternatives | 2285 | 1651 | 72.3 | 634 | 27.7 |
| Non-alcoholic beverages | 2925 | 2274 | 77.7 | 651 | 22.3 |
| Sauces, dressings, spreads and dips | 2868 | 2040 | 71.1 | 828 | 28.9 |
| Seafood and seafood products | 842 | 625 | 74.2 | 218 | 25.9 |
| Snack foods | 1284 | 1054 | 82.1 | 230 | 17.9 |
| Sugars, honey and related products | 541 | 269 | 49.7 | 272 | 50.3 |
